# Malignant hepatic epithelioid angiomyolipoma with recurrence in the lung 7 years after hepatectomy: a case report and literature review

**DOI:** 10.1186/s40792-016-0158-1

**Published:** 2016-04-02

**Authors:** Yasunari Fukuda, Hideyasu Omiya, Koji Takami, Kiyoshi Mori, Yoshinori Kodama, Masayuki Mano, Yoriko Nomura, Jun Akiba, Hirohisa Yano, Osamu Nakashima, Mitsumasa Ogawara, Eiji Mita, Shoji Nakamori, Mitsugu Sekimoto

**Affiliations:** Department of Surgery, National Hospital Organization Osaka National Hospital, Osaka, Japan; Department of Pathology, National Hospital Organization Osaka National Hospital, Osaka, Japan; Department of Gastroenterological Surgery, Kurume University School of Medicine, Kurume, Japan; Department of Pathology, Kurume University School of Medicine, Kurume, Japan; Department of Clinical Laboratory Medicine, Kurume University Hospital, Kurume, Japan; Department of Respiratory Medicine, National Hospital Organization Osaka National Hospital, Osaka, Japan; Department of Gastroenterology and Hepatology, National Hospital Organization Osaka National Hospital, Osaka, Japan; Department of Gastroenterological Surgery, Osaka University Graduate School of Medicine, 2-2 Yamadaoka, Suita, 565-5111 Osaka, Japan

**Keywords:** Hepatic angiomyolipoma, Perivascular epithelioid cell, Malignant potential

## Abstract

Angiomyolipoma (AML) arising in the liver is rare and usually benign, but it occasionally has malignant potential. A 58-year-old man with a liver tumor identified by a previous doctor with features suggestive of hepatocellular carcinoma on computed tomography (CT) underwent anterior segmentectomy of the liver in 2006. Microscopically, the tumor was composed of exclusively epithelioid cells that were scatteredly positive for human melanoma black 45 on immunohistochemistry. Accordingly, primary hepatic epithelioid AML (eAML) was diagnosed. The patient was subsequently referred to our hospital for follow-up after hepatectomy. He had event-free survival for nearly 7 years. In 2013, two well-defined round nodules were detected in the right lung field by chest CT, and partial pneumonectomy was performed for diagnosis and treatment. Histological examination of the resected lung tissue showed that it was morphologically and immunohistochemically identical to his primary hepatic eAML, leading to the diagnosis of pulmonary metastasis. This paper demonstrates a rare case of malignant hepatic eAML with late recurrence in the lung after hepatectomy.

## Background

Angiomyolipoma (AML) is a relatively rare mesenchymal tumor. Histologically, it is composed of blood vessels, adipose tissue, and smooth muscle. AML is most commonly observed in the kidney, followed by the liver. The tumor is thought to originate from pluripotent perivascular epithelioid cells (PECs), although its normal tissue counterpart remains unclear [1, 2]. PEC is immunopositive for melanocytic and myogenic markers [3]. In addition, a group of tumors formed by PEC proliferation has been recognized as PEComas, firstly described by Bonetti and colleagues in 1992 [1] and currently recognized by the World Health Organization [4]. AML is considered a member of the PEComa family [4]. AML can be histologically classified according to the ratio of the three heterogeneous components, from the classic triphasic type to a monotypic type [5]. Among the monotypic types of AML, epithelioid AML (eAML) is predominantly composed of epithelioid cells and does not contain blood vessels or adipose tissue. Although in general AML has good biological behavior, it can have malignant potential with distant metastasis, recurrence, and associated mortality [6–15]. We describe a case of malignant hepatic eAML that recurred in the lung nearly 7 years after hepatectomy for the initial tumor and review the characteristics of malignant hepatic AML with recurrence.

## Case presentation

**Fig. 1:**
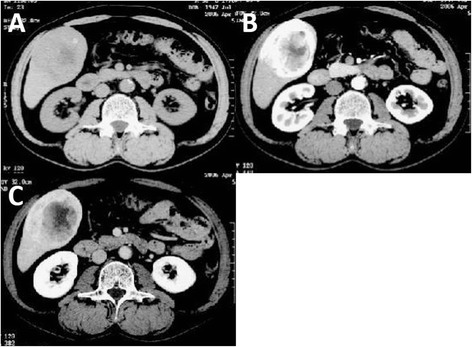
Imaging of primary hepatic AML. **a** Plain CT showed a low-density mass in segment 5 of the liver. **b**, **c** Enhanced CT showed a tumor with hemorrhagic and necrotic changes, with early arterial phase staining and late phase washout

**Fig. 2:**
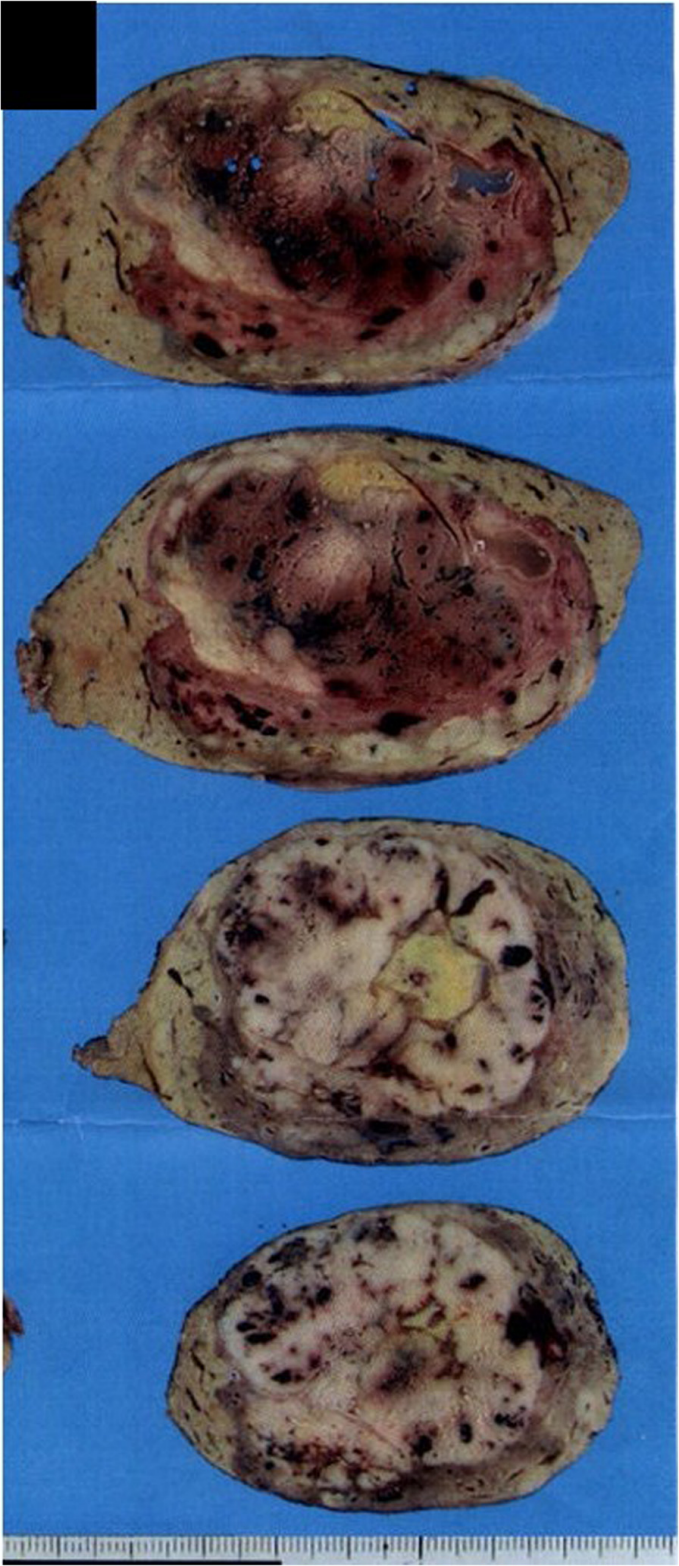
Sections of the resected hepatic AML specimen. The tumor included a white to yellow solid mass with areas of hemorrhage

**Fig. 3:**
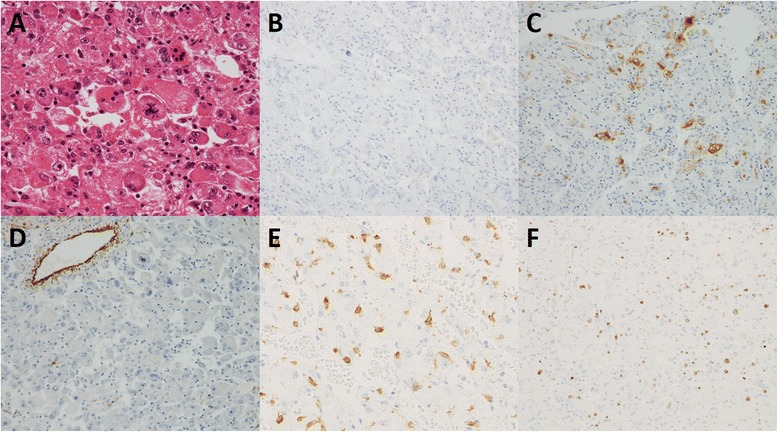
Pathological findings of hepatic AML. **a** Hematoxylin and eosin staining showed atypical epithelioid cells containing clear to eosinophilic granular cytoplasm and pleomorphic nuclei (original magnification ×400). **b** Immunohistochemical staining was negative for HepPar 1 (original magnification ×200), **c** scatteredly positive for HMB-45 (original magnification ×200), **d** inconspicuous for SMA (original magnification ×200), and **e** negative for c-kit (mast cells were expressing c-kit; original magnification ×400). **f** The Ki-67 labeling index was 12.9 % (original magnification ×200)

A 58-year-old man was incidentally found to have a liver tumor measuring 20 mm by abdominal ultrasonography during a routine company health examination in 2004. The tumor grew in size over the next 2 years, and closer inspections were conducted by a previous physician in 2006. Enhanced computed tomography (CT) of the abdomen showed a 70-mm mass with hemorrhagic and necrotic changes in the right lobe of the liver (segment 5), with early arterial phase staining and late phase washout, which seemed to be consistent with hepatocellular carcinoma (HCC) (Fig. 1). Anterior segmentectomy of the liver was performed. Macroscopically, non-encapsulated tumor had irregular borders. Sectioning of resected specimen revealed a white to yellow, friable, hemorrhagic mass measuring 63 × 50 mm (Fig. 2). Microscopically, hematoxylin and eosin staining showed atypical epithelioid cells containing abundant clear to eosinophilic granular cytoplasm arranged in nests and sheets. The cells were unevenly distributed and had pleomorphic nuclei of varying sizes. Blood vessels were inconspicuous and mature adipose tissue was not detected. Both bile ducts and portal tracts were involved in the tumor. Immunohistochemically, the tumor cells were negative for hepatocyte paraffin 1 (HepPar 1) and alpha fetoprotein. In contrast, the tumor cells were scatteredly positive for melanocytic markers including human melanoma black 45 (HMB-45) and Melan A, but negative for smooth muscle antigen (SMA) and S100 protein, together with negative for c-kit and moderately high Ki-67 labeling index (12.9 %) (Fig. 3). Accordingly, primary hepatic eAML was diagnosed at that time.

**Fig. 4:**
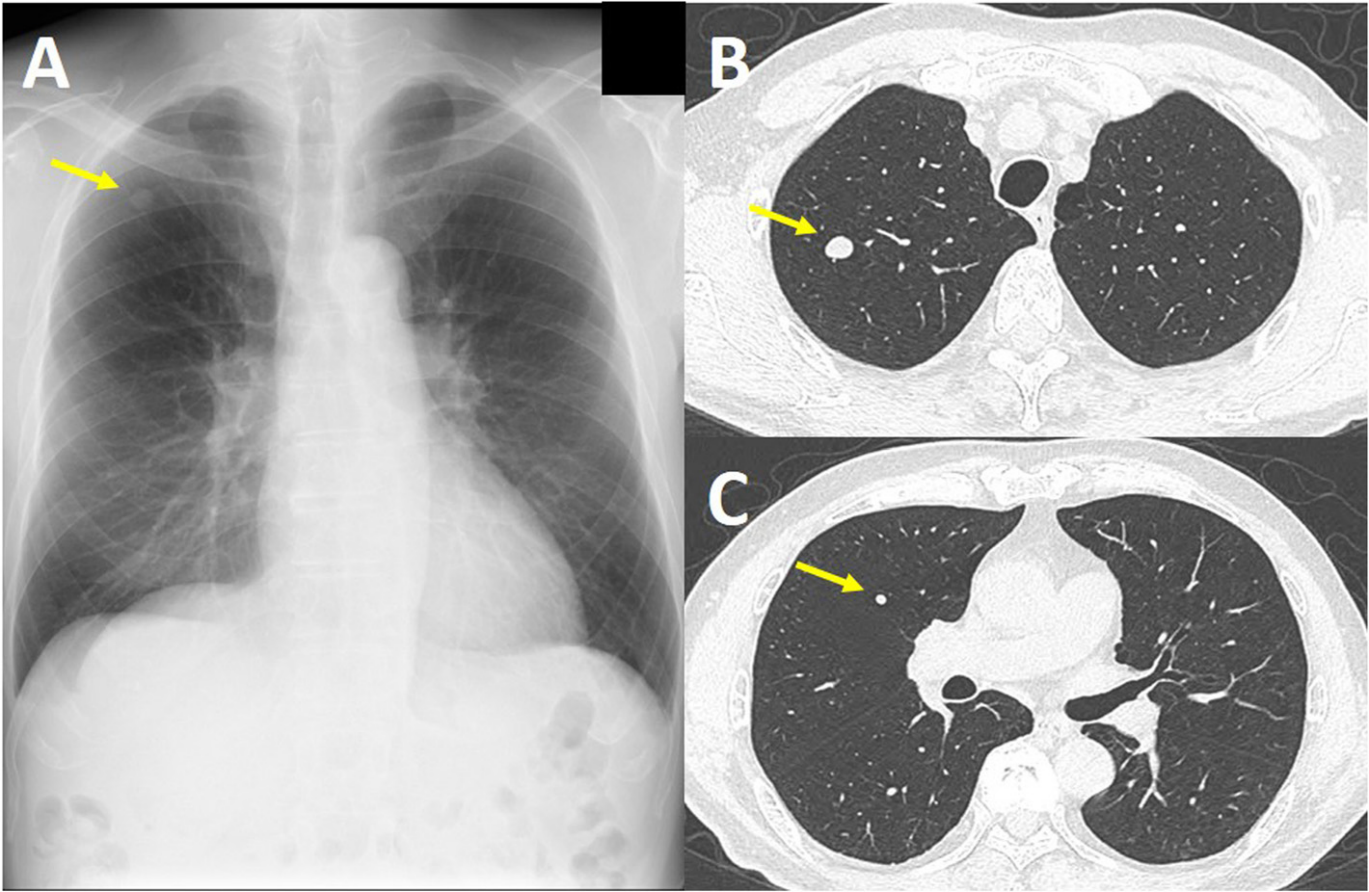
Imaging of the recurrent lesions in the lung. **a** Chest X-ray demonstrated a well-defined nodule in the right upper lung field (*arrow*). **b**, **c** Chest CT demonstrated two round nodules in segments 1 and 3 of the right lung, respectively (*arrow*)

**Fig. 5:**
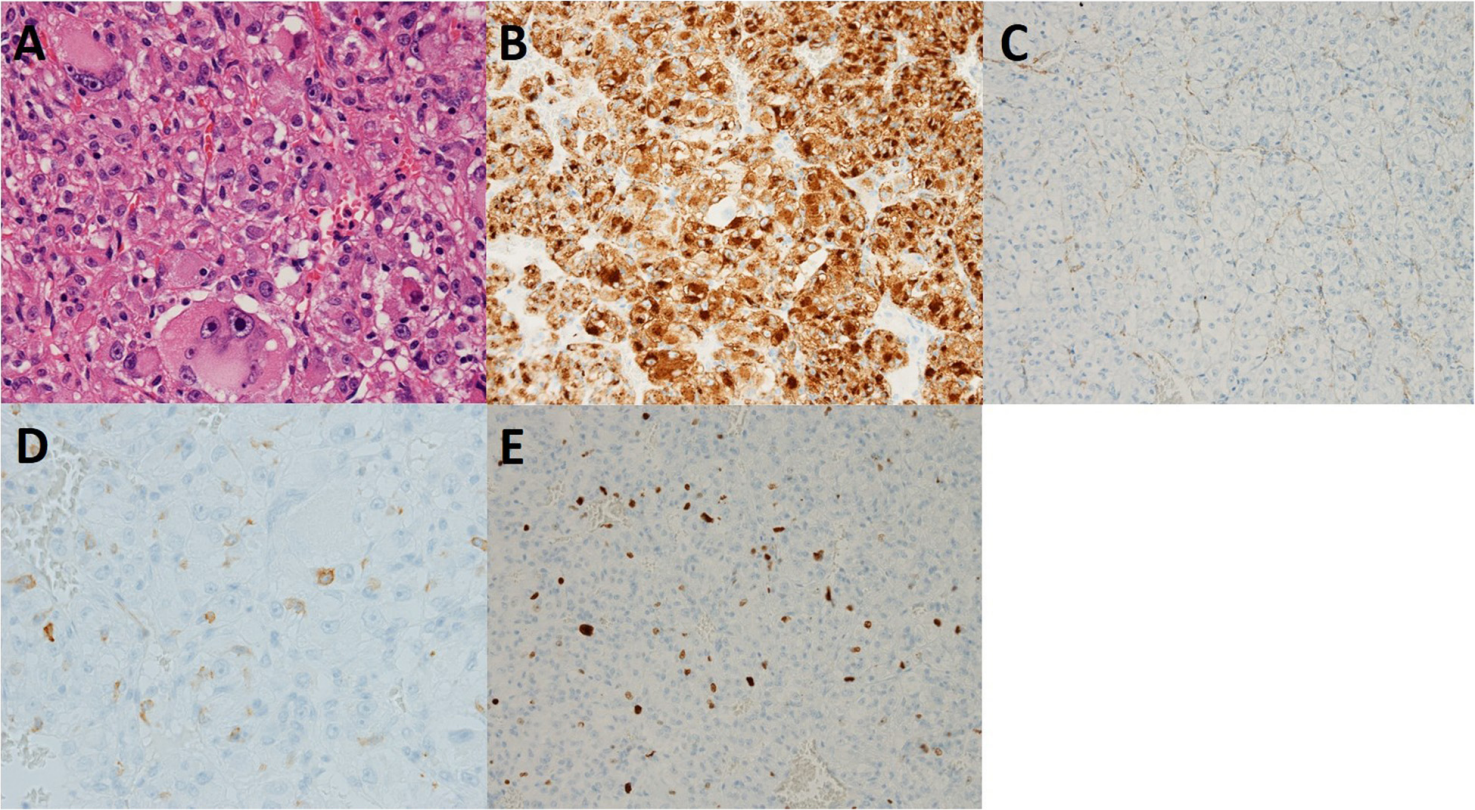
Pathological findings of the recurrent lesions in the lung. **a** Hematoxylin and eosin staining showed that the tumor cells were morphologically similar to those of the primary hepatic AML tumor (original magnification ×400). **b** Immunohistochemical staining was diffusely positive for HMB-45 (original magnification ×200), **c** negative for SMA (original magnification ×200), and **d** negative for c-kit (original magnification ×400). **e** The Ki-67 labeling index was 9.0 % (original magnification ×200)

After hepatectomy, the patient was referred to our hospital and followed every 6 months on an outpatient basis. No hepatic recurrence was observed over nearly 7 years. In 2013, a chest X-ray showed a well-defined nodule in the right lung field (Fig. 4a). Chest CT showed two round nodules in segments 1 and 3 of the right lung, measuring 10 and 4 mm, respectively (Fig. 4b, c). Fluorodeoxyglucose-positron emission tomography did not show any uptake. Since the diagnosis could not be confirmed by inspection through bronchoscopy, partial pneumonectomy was performed for diagnostic and therapeutic purposes. Macroscopically, the two resected specimens included solid white masses. Microscopically, the neoplastic cells were morphologically similar to those of the primary hepatic tumor. There was no evidence of vascular invasion. Immunohistochemically, the tumor cells were diffusely positive for HMB-45 and negative for SMA and S-100 protein. In addition, the tumor cells were negative for c-kit and the Ki-67 labeling index was 9.0 % (Fig. 5). Therefore, a diagnosis of lung metastases from hepatic eAML was made. Loss of tuberous sclerosis complex (TSC) genes, *TSC1* and *TSC2*, was not detected. The patient has remained recurrence-free on a closer follow-up schedule for 2 years after pneumonectomy.

### Discussion

Since it was first described by Ishak [16] in 1976, hepatic AML is now a well-recognized tumor. The number of reports on hepatic AML, including malignant cases, has continued to increase worldwide. In recent years, the clinicopathological characteristics of malignant hepatic AML have been intensively investigated despite many challenges. Tumor size has been reported as a simple and clear predictor of malignant AML [10–14, 17]. Although the cutoff value varies in the literature, Ding and colleagues [14] identified that tumors greater than 60 mm in diameter might have malignant potential and this was true of our case (63 mm). In terms of histology, cytologic atypia, pleomorphism, necrosis, and high mitotic rate were reported as predictors of malignant potential [10, 11, 13, 17]. The presence of cytologic atypia and necrosis are frequently observed in malignant cases, including ours. However, Nonomura and colleagues [5] demonstrated that atypia and pleomorphism are occasionally found in epithelioid cells and does not always indicate malignant hepatic AML. In addition, the monotypic epithelioid variant of AML arising from the kidney had been regarded as having malignant potential [18, 19], whereas data on those of hepatic origin are scarce. Recently, the number of reports on malignant classical AML in the liver has also been increasing [10, 12, 13, 15]. Accumulation of cases is necessary to clarify the histological differences between malignant and benign AML.

Immunohistochemically, there was a difference in the degree of staining for HMB-45 between primary and metastatic lesion. Considering intratumoral heterogeneity has been observed in various malignancies [20], this difference may suggest malignant potential in our case. Moreover, a previous investigation showed that lack of c-kit expression is suggestive of malignant hepatic AML, [13] in contrast to c-kit immunopositivity in benign lesions [21]. Indeed, in four cases where c-kit expression was evaluated, all were negative or weakly positive. Although further investigation of the intensity of c-kit staining in malignant cases is required, it is potentially a predictive marker.

There is a paucity of detailed data on the metastasis or recurrence pattern of malignant hepatic AML. As summarized in Table 1, the most frequently observed site of recurrence is the liver (nine cases), followed by the lung (three cases), pancreas (two cases), and other organs, indicating a hematogenous pattern of metastasis for malignant hepatic AML. Furthermore, our case showed that the lung could be the first site of recurrence for malignant hepatic AML. Thus, prudent evaluation of the lung fields by chest X-ray or chest CT in addition to following the residual liver should be conducted.

**Table 1:** Previous reports of malignant hepatic AML with recurrence

Case	Reference	Age	Gender	Size (cm)	Resection for primary	Histology	Cytologic atypia	Necrosis	Ki-67	c-kit	Recurrence site	RFS (months)	Resection for recurrence	Prognosis
1	[7]	70	F	15	+	Epithelioid variant	+	+	N/A	N/A	Liver	5	−	Alive with the disease details were not shown
2	[8]	16	F	12	+	Epithelioid variant	+	N/A	N/A	N/A	Liver	6	+	N/A
3	[9]	51	F	10	+	Epithelioid variant	N/A	N/A	2 %	N/A	Liver	36	+	Alive without the disease 1.5 years after recurrence
4	[10]	30	M	18	+	Classical AML	+	+	30 %	Weakly +	Liver, pancreas, and lung	36	+	Died 4 months after recurrence
5	[11]	60	F	14	+	Epithelioid variant	−	−	N/A	Weakly +	Liver, trapezius muscle, bladder, lung, and pancreas	108	+	Alive with the disease details were not shown
6	[12]	37	F	13	+	Classical AML	+	N/A	N/A	N/A	Liver, lung	6	−	Died 8 months after recurrence
7	[13]	43	F	11	+	Classical AML	+	+	Weak	−	Liver, peritonium, retroperitonium, and omentum	6	−	Died 3 months after recurrence
8	[14]	31	F	8	+	N/A	N/A	N/A	N/A	N/A	Liver	72	−	Died 1 year after recurrence
9	[15]	37	F	9	+	Classical AML	+	+	4 %	N/A	Liver	36	+ (transplantation)	Alive without the disease details were not shown
Present case		58	M	6.3	+	Epithelioid variant	+	+	13 %	Weakly +	Lung	84	+	Alive without the disease 2 years after recurrence

*AML* angiomyolipoma, *RFS* recurrence-free survival, *N/A* not available

Four patients died due to disease recurrence, indicating an unfavorable prognosis after recurrence [10, 12–14]. In a case described by Dalle and colleagues [7], the tumor was observed for 5 years before it was resected, and multiple metastases appeared in the residual liver just 5 months after resection. Moreover, median recurrence-free survival of the ten patients with recurrence was 36 months (range, 5–108 months); three patients relapsed more than 5 years after initial resection. Prompt surgical treatment and careful follow-up for a long period is crucial to increasing the survival of patients with malignant disease. In addition, our case suggests that early detection and re-resection of the site of recurrence could improve the prognosis of patients with malignant case.

## Conclusions

In conclusion, we reported a case of malignant hepatic AML with late recurrence in the lung and examined the characteristics of recurrence in patients with malignant hepatic AML. The prognosis of patients with malignant hepatic AML is poor. In this respect, we regard hepatic AML as a borderline malignant tumor, and aggressive therapeutic intervention is recommended since surgical resection is indisputably the most reliable curative treatment.

## Consent

Written informed consent was obtained from the patient for publication of this case report and any accompanying images. A copy of the written consent is available for review by the Editor-in-Chief of this journal.

## Abbreviations

AML: angiomyolipoma
CT: computed tomography
eAML: epithelioid AML
HCC: hepatocellular carcinoma
HepPar 1: hepatocyte paraffin 1
HMB-45: human melanoma black 45
PEC: perivascular epithelioid cell
SMA: smooth muscle antigen
TSC: tuberous sclerosis complex
